# Ultrasound-Assisted Extraction of *Pleurotus ostreatus* By-Products for the Development of Nutritionally Enhanced Gluten-Free Cookies: Nutritional, Sensory, and Metabolomic Insights

**DOI:** 10.3390/antiox15101347

**Published:** 2026-10-19

**Authors:** Jhazmin Quizhpe, Rocío Peñalver, Pablo Ayuso, Leilei Zhang, Pascual García-Pérez, Luigi Lucini, Gema Nieto

**Affiliations:** 1 Department of Food Technology, Food Science and Nutrition, Faculty of Veterinary Sciences, Regional Campus of International Excellence “Campus Mare Nostrum”, University of Murcia, Espinardo, 30100 Murcia, Spain; jhazminedith.quizhper@um.es (J.Q.); rocio.penalver@um.es (R.P.); pablo.ayuson@um.es (P.A.); pascual.garcia@um.es (P.G.-P.); 2 Department for Sustainable Food Process, Università Cattolica del Sacro Cuore, Via Emilia Parmense 84, 29122 Piacenza, Italy; leilei.zhang@unicatt.it (L.Z.); luigi.lucini@unicatt.it (L.L.)

**Keywords:** functional foods, bioactive compounds, mushroom by-products, metabolomics, sustainable food processing, UHPLC-HRMS

## Abstract

The growing demand for gluten-free (GF) products has highlighted nutritional limitations in commercial bakery products, particularly low fiber, mineral, and bioactive compound content. This study evaluated the valorization of *Pleurotus ostreatus* by-products as nutritionally valuable ingredients for GF cookies, focusing on the nutritional and metabolomic effects of ultrasound-assisted extraction (UAE). Untreated and UAE-treated by-products were incorporated into GF cookies at 4% and 8% (*w*/*w*). Proximate composition, mineral content, antioxidant capacity, untargeted phenolic profile, and sensory characteristics were assessed. UAE modified the nutritional and metabolomic profiles of mushroom by-products, increasing soluble dietary fiber, protein, and essential minerals such as Zn, Cu, and Mn. Untargeted metabolomic analysis revealed that UAE selectively promoted low-molecular-weight phenolic acids. Compared with a commercial GF reference, reformulated cookies exhibited higher protein and total dietary fiber contents, fulfilling the “high fiber” nutritional claim, together with a more balanced mineral profile. All reformulated cookies also showed higher iron content than the commercial GF reference, allowing the “source of iron” nutritional claim. Metabolomic profiling confirmed greater phenolic diversity and abundance in reformulated cookies, while UAE-treated by-products enhanced phenolic acids. Overall, UAE-treated mushroom by-products represent a clean-label strategy to enhance the nutritional quality and bioactive compound profile of GF bakery products.

## 1. Introduction

Celiac disease (CD) is an autoimmune enteropathy triggered by dietary gluten consumption, leading to villous atrophy and subsequent malabsorption of key nutrients such as iron, calcium, folate, and B-complex vitamins [1]. Globally, CD prevalence is estimated at approximately 0.7–1.4% of the population, with many cases remaining undiagnosed [2]. Currently, a strict gluten-free (GF) diet remains the only effective therapeutic strategy. Beyond those diagnosed with CD, a growing number of consumers, motivated by non-celiac sensitivities to gluten or wheat or by perceived health benefits, voluntarily adopt GF diets [3,4]. This trend has increased consumer demand for GF products, contributing to sustained global market expansion, which has reported a compound annual growth rate of 7.6% between 2020 and 2027 [5].

However, recent studies have shown that GF bakery products exhibit remarkable nutritional limitations, presenting increased levels of sugars and calories, as well as reduced protein, dietary fiber, and bioactive compounds content [6,7]. To overcome these limitations, reformulation strategies have focused on enhancing their nutritional quality by incorporating pseudocereal and legume flours such as those derived from chickpea, quinoa, teff, and amaranth [8,9] as well as nutritionally valuable plant-derived ingredients, including moringa and agro-industrial by-products from processing broccoli and orange [10,11,12]. Consequently, the valorization of agro-industrial by-products has emerged as a promising strategy to reduce waste and generate value-added ingredients for the development of new food products with enhanced nutritional and bioactive profiles [13,14].

In this context, by-products generated during the cultivation and processing of oyster mushrooms (*Pleurotus ostreatus*), such as stipes, mycelium, and spent mushroom substrate, stand out for their high content of β-glucans, polyphenols, proteins, essential minerals, and dietary fiber [15]. These compounds confer a wide range of biological activities, including antioxidant, antihyperlipidemic, antitumor, immunoregulatory, and bacteriostatic effects [16,17]. According to the Food and Agriculture Organization of the United Nations (FAO) [18], global production of mushrooms and truffles exceeded 50 million tons in 2023, with the genus *Pleurotus* spp. representing around 27% of this volume [19]. Consequently, the edible mushroom industry generates substantial amounts of residual biomass, estimated at roughly 5 kg of wet by-product per kg of fresh mushrooms processed [20]. In addition, emerging non-thermal technologies can further modify the bioactive compound profile of these by-products, representing a promising strategy to improve the sustainability of food systems. Among them, ultrasound-assisted extraction (UAE) stands out as an environmentally friendly technique that can enhance bioactive compound recovery while preserving nutritional quality. Ultrasound treatment (above 20 kHz) induces acoustic cavitation, promoting cell membrane disruption and facilitating the release of bioactive compounds into the solvent [21]. This mechanism not only accelerates extraction compared to conventional methods but also reduces solvent consumption and energy requirements, aligning with the principles of sustainable processing. In addition, this technique has demonstrated remarkable versatility across different food matrices, including the extraction of natural pigments, phenolic compounds, and pectins from fruit and vegetable by-products [22,23,24]. Therefore, applying UAE to *Pleurotus* by-products represents a promising strategy to obtain bioactive compound-enriched extracts that can be incorporated into nutritionally enhanced food formulations.

In parallel, metabolomics combined with chemometric tools offers a comprehensive framework to characterize the molecular complexity of food matrices and their derived ingredients [25]. Advanced mass spectrometry coupled with multivariate analysis enables the identification of a broad range of metabolites, including phenolic acids and lipid derivatives, with high sensitivity and accuracy. Chemometric approaches, such as principal component analysis (PCA) and partial least squares discriminant analysis (PLS-DA), allow the interpretation of complex datasets, identifying compositional shifts associated with processing and formulation [26,27]. Applied to GF bakery products, metabolomics is particularly valuable for understanding how novel ingredients influence metabolite composition, antioxidant capacity, and other quality attributes, thereby supporting the development of more nutritionally balanced and bioactive compound-enriched products.

The present study aimed to assess the effect of ultrasound-assisted extraction (UAE) applied to mushroom by-products and their subsequent incorporation into GF cookie formulations. Furthermore, comprehensive metabolomic characterization of the final products was performed to elucidate compositional changes and evaluate the potential of these by-products as nutritionally enhanced and bioactive compound-enriched ingredients. This approach supports the valorization of mushroom by-products through sustainable extraction technologies and the development of nutritionally improved and more sustainable food products.

## 2. Materials and Methods

### 2.1. Preparation and Treatment of Pleurotus ostreatus By-Products

The vegetable materials (Oyster mushroom (*Pleurotus ostreatus*) by-products, primarily composed of mycelium and stipe residues, were washed, cut into uniform 10 mm^3^ cubes, and dried in a forced-air oven at 60 °C for 24 h. The dehydrated residue was milled and sieved through a 300 µm mesh to obtain the mushroom control powder (MC), consisting of the whole dried mushroom by-products.

Ultrasound-assisted extraction (UAE) was conducted using an ultrasonic processor (UIP500hdT, Hielscher Ultrasonic GmbH, Teltow, Germany) operating at 20 kHz and 185 W for 5 min. The extraction conditions were selected based on previous studies on the UAE of bioactive compounds from *Pleurotus ostreatus* and related mushroom matrices, as summarized by Ayuso et al. [15]. The MC was mixed with a 40:60 (*v*/*v*) ethanol–water solution at a solid-to-liquid ratio of 1:10 (*w*/*v*) and a limiting sonication temperature of 45 °C. After sonication, the mixture was filtered through a 200 µm mesh to separate the solid residue. Ethanol was removed under reduced pressure at 40 °C using a rotary evaporator (Hei-VAP Core, Heidolph Instruments, Schwabach, Germany). The resulting aqueous extract was subsequently freeze-dried and sieved to obtain MU, a fine powder corresponding to the dried UAE extract. Therefore, MU represented the fraction of the by-product recovered in the extract after UAE and subsequent solvent removal and freeze-drying. Before their incorporation into the GF cookie formulations, MC and MU were characterized in terms of their nutritional composition and physicochemical properties as described in the following sections.

### 2.2. Gluten-Free Cookies Formulation

All ingredients used to prepare the GF cookies were obtained from a local supermarket (Murcia, Spain) and combined according to the formulations shown in Table 1. Dry ingredients were first mixed manually until homogeneous. Then olive oil, beaten eggs, and milk were gradually incorporated to obtain a uniform dough. The dough was allowed to rest at room temperature for 10 min, rolled to a thickness of approximately 5 mm, cut into circular pieces (4 cm diameter), and baked at 180 °C for 15 min. Five different cookie formulations were prepared. The control formulation (CC) contained no mushroom by-products. The remaining formulations were supplemented with MC or MU at inclusion levels of 4% and 8% (*w*/*w*), expressed as a percentage of the total flour weight, resulting in formulations C-4MC, C-8MC, C-4MU, and C-8MU. These inclusion levels were selected based on previous studies demonstrating that mushroom powder can be incorporated into cookie formulations within this range to improve nutritional quality while maintaining acceptable sensory properties [28,29]. Both MC and MU were incorporated as additional ingredients and did not replace any component of the original formulation. After baking, the cookies were cooled at room temperature. Half of the samples were stored in cotton cloth bags for sensory analysis and color parameter testing the following day. The other half of the samples were ground, vacuum-packed in polyethylene bags, and stored at −20 °C until further analysis.

**Figure 1:**
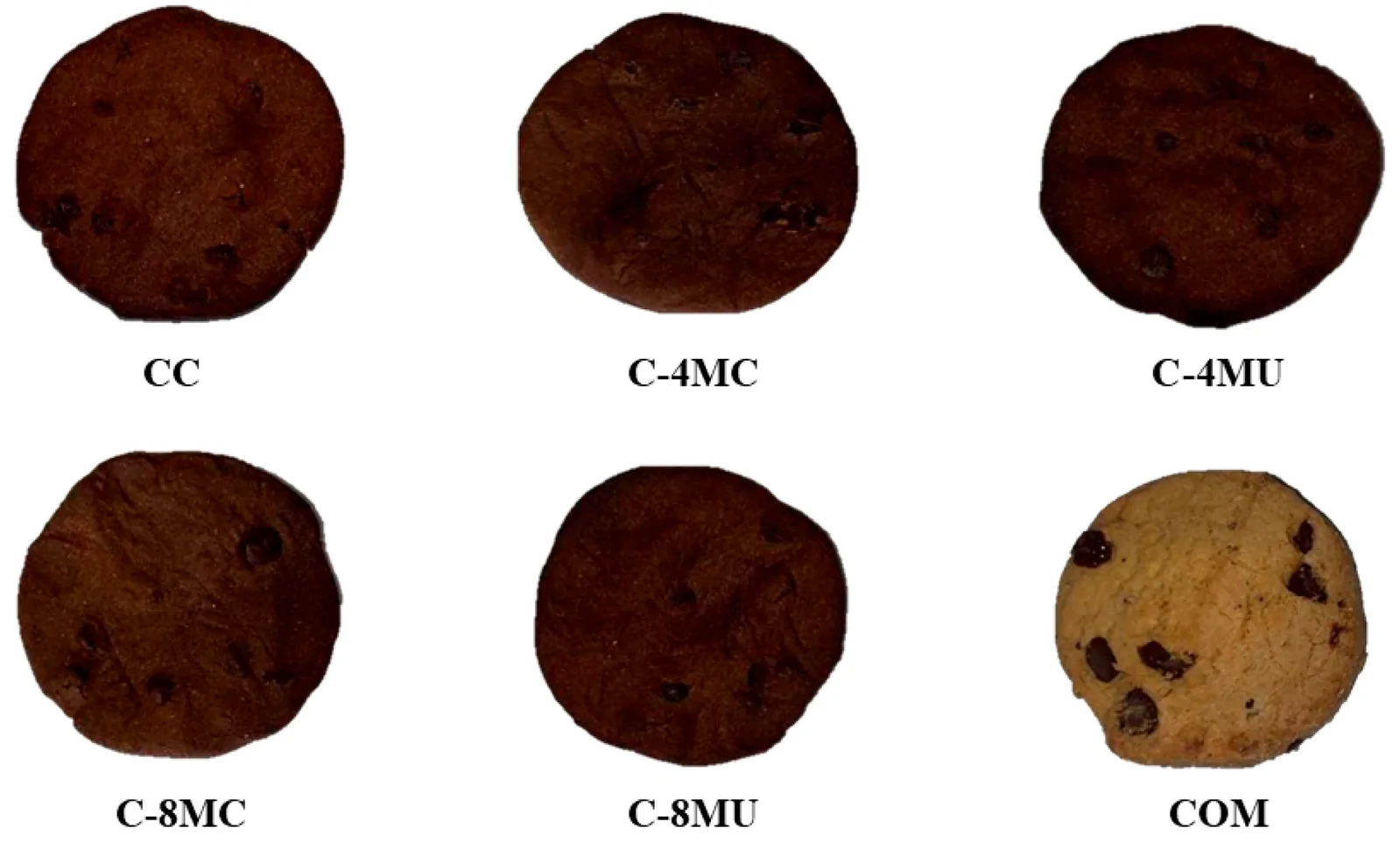
Visual appearance of the different gluten-free cookie formulations. Abbreviations: CC: gluten-free control cookie; C-4MC: gluten-free cookie with 4% MC; C-4MU: gluten-free cookie with 4% MU; C-8MC: gluten-free cookie with 8% MC; C-8MU: gluten-free cookie with 8% MU; COM: commercial gluten-free cookie.

For comparative purposes, a commercial cookie (COM) was also analyzed. The selected product was one of the few gluten-free chocolate chip cookies readily available in local supermarkets, representing a commonly marketed product within this category. According to the label, its composition includes chocolate chips, sunflower lecithin, vanilla flavor, corn flour, margarine (non-hydrogenated vegetable oil and fat, sunflower oil, coconut fat, water), sugar, rice flour, pasteurized egg, tapioca starch, salt, and thickeners (guar gum, xanthan gum). The visual appearance of the GF cookies is presented in Figure 1.

### 2.3. Proximate Composition

Proximate composition of the mushroom by-products and GF cookies was assessed in accordance with the Association of Official Analytical Chemists official methods [30]. Determination of total fat (920.39), protein (955.04), ash (923.03), moisture (964.22), insoluble, soluble, and total dietary fiber (991.43) was performed using three independently prepared samples for each formulation (*n* = 3). Total carbohydrate and energy contents were determined in accordance with Food and Agriculture Organization of the United Nations (FAO) guidelines [31].

The quantification of macro- and micro-mineral elements in both mushroom by-products and GF cookies was carried out through inductively coupled plasma optical emission spectrometry (ICP-OES) employing a Thermo iCAP 6500 Duo instrument (Thermo Fisher Scientific, Waltham, MA, USA) [32]. Prior to analysis, 0.10 g of each solid sample was digested with 4 mL of concentrated nitric acid and 1 mL of 33% hydrogen peroxide using a microwave digestion system. The samples were heated to 220 °C for 20 min following a 30 min temperature ramp. After digestion, the samples were diluted to 25 mL with ultrapure water. A reagent blank and a control sample were processed together with the samples. Calibration and analytical controls were prepared from a commercial multielement standard solution. The iTEVA software (version 5, Thermo Fisher Scientific) was used for processing analytical data, and the concentrations of each element were reported as g/100 g and mg/kg for macro- and micro-minerals, respectively. Three independently prepared samples were analyzed for each formulation (*n* = 3).

### 2.4. Antioxidant Capacity and Total Phenolic Content (TPC) Determination

Mushroom by-products and GF cookies were subjected to bioactive compound extraction before analysis. Two grams of each fresh sample were homogenized with 8 mL of methanol/water (80:20, *v*/*v*) solution, incubated in darkness at 4 °C for 24 h, and then centrifuged at 4500 rpm for 25 min. The resulting supernatants were filtered through 0.45 µm nylon membranes. The antioxidant capacity of mushroom by-products and GF cookies was evaluated using the Ferric Reducing Antioxidant Power (FRAP) assay, the 2,2′-azino-bis (3-ethylbenzothiazoline-6-sulfonic acid) cation radical (ABTS) assay, and the 2,2-diphenyl-1-picrylhydrazyl (DPPH) radical scavenging activity. For the FRAP assay, 100 µL of the extracted sample was combined with 1 mL of FRAP reagent for 4 min under dark conditions, and then the absorbance was measured at 593 nm [33]. The ABTS assay was conducted following the protocol described by Re et al. [34] by incubating 100 µL of the extracted sample with the ABTS reagent for 4 min and measuring absorbance at 734 nm. The DPPH radical scavenging activity was determined by adding 3.9 mL of a methanolic DPPH solution to 100 µL of the sample and measuring absorbance at 515 nm after 30 min in dark conditions [35]. Antioxidant capacity results were reported as µmol Trolox equivalents (TE) per 100 g of sample, using Trolox as the positive control for the calibration curve. Finally, the total phenolic content (TPC) of mushroom by-products and GF cookies was quantified by measuring the absorbance at 750 nm after 1 h incubation of 100 µL of the extracted samples with 400 µL of sodium carbonate (Na_2_CO_3_) and 500 µL of Folin–Ciocalteu reagent [36]. The results were expressed as mg gallic acid equivalents (GAE) per 100 g of sample, using gallic acid as the standard. For each sample, three independent replicates were analyzed (*n* = 3). Spectrophotometric measurements were carried out using an Evolution 300 spectrophotometer (Thermo Scientific, Waltham, MA, USA).

### 2.5. Physicochemical Parameters

Color parameters were measured with a CR-400 portable colorimeter (Konica Minolta, Tokyo, Japan), previously calibrated with a standard white plate provided by the manufacturer. Lightness (L*), chroma (*C*), hue (*h*), redness (a*), and yellowness (b*) parameters were measured in accordance with the CIEL*a*b* color space system [37]. The total color difference (∆E*) of each formulation compared to the control cookie (CC) was calculated with the following formula:

$${∆E}^{*}=\sqrt{{\left(L_{s}^{*}-L_{c}^{*}\right)}^{2}+{\left(a_{s}^{*}-a_{c}^{*}\right)}^{2}+{\left(b_{s}^{*}-b_{c}^{*}\right)}^{2}}$$

with the subscript s indicating the value for the sample and c for the control.

The pH of GF cookies was determined by a sensION+ PH31 pH meter (Hach-Lange, Barcelona, Spain) after homogenizing 10 g of fresh samples with 90 mL of distilled water at room temperature. Subsequently, total titratable acidity (TTA) was assessed by titration with 0.1 N sodium hydroxide (NaOH) to an endpoint pH of 8.5. Results were expressed as mL NaOH/10 g sample. Three independently prepared samples were used as experimental replicates for each sample type and formulation (*n* = 3).

### 2.6. Profiling Phenolic Compounds by Untargeted Metabolomics

For the untargeted metabolic characterization, samples of GF cookies and mushroom by-products were lyophilized using a LyoEpic-85 system (Coolvacuum Technologies S.L., Barcelona, Spain). For each experimental group, three independent samples were weighed in duplicate (0.5 g) and processed separately through the extraction procedure. Each freeze-dried sample was extracted with 5 mL of 80% aqueous methanol with 0.1% formic acid (*v*/*v*). Subsequently, the mixes were subjected to ultrasound-assisted extraction for 10 min to enhance compound release, followed by centrifugation at 8000× *g* for 10 min at 4 °C (OHAUS FC5718R, OHAUS Corp., USA). The supernatants were filtered through 0.22-μm syringe filters, yielding six independent replicates per experimental group (*n* = 6). In parallel, pooled quality control (QC) was prepared by combining 20-μL aliquots from all individual samples.

The phenolic profiling was acquired by ultra-high-performance liquid chromatography coupled to high-resolution mass spectrometry (UHPLC-HRMS). The chromatography stage was performed on a 1290 UHPLC system (Agilent Technologies^®^, Santa Clara, CA, USA) including a PFP reversed-phase column (2.1 mm × 100 mm, 1.9 μm particle size). A gradient elution was employed, consisting of water (solvent A) and acetonitrile (solvent B), both containing 0.1% (*v*/*v*) formic acid. Over 32 min, solvent A was linearly decreased from 96% to 6%, while the flow rate was maintained at 200 μL min^−1^, injecting 6 μL of sample volume. Detection by mass spectrometry was carried out on a G6550 quadrupole-time-of-flight (QTOF) system equipped with a JetStream electrospray ionization source (Agilent^®^), operating in positive ion mode (ESI+) with a scan range of 100–1200 *m*/*z*. Nitrogen was used as both sheath (12 L min^−1^, 315 °C) and drying gas (14 L min^−1^, 250 °C), while the nebulizer pressure was set at 310.3 kPa. Nozzle and capillary voltages operated at 350 V and 4000 V, respectively, as previously optimized [38]. Samples were acquired in MS-only mode, and the QC samples were acquired using the data-dependent (autoMSMS) mode. Data processing was conducted employing MassHunter Profinder software (Agilent^®^), with the find-by-formula algorithm for metabolite annotation. Feature alignment was achieved by applying post-acquisition filters, based on monoisotopic mass, isotopic ratio, and isotopic spacing, considering the possible adducts, as previously described [39]. Compounds detected in fewer than five of six replicates per group were excluded. Compound annotation was carried out following Level 2 confidence criteria of the COSMOS (Coordination of Standards in Metabolomics) initiative, using a customized database that included phenolic compounds and their metabolites compiled from Phenol-Explorer (version 3.60) [39]. Annotation followed Level 2 standards of the COSMOS metabolomics initiative, based on an in-house curated database of phenolic compounds.

### 2.7. Sensory Analysis

A hedonic sensory analysis was conducted to assess the organoleptic characteristics of the different GF cookies. An untrained panel of 40 consumers, aged between 19 and 63 years of both genders, evaluated sensory attributes of the different samples using a 5-point hedonic scale ranging from 1 (extremely dislike) to 5 (extremely like). The sensory attributes evaluated included appearance, aroma, texture, taste, color, overall acceptability, and purchase intention. The sensory evaluations were performed in individual sensory booths according to ISO guidelines [40]. Additionally, mineral water was provided for mouth rinsing between samples. Each panelist evaluated six samples (one from each formulation and the commercial) in a single session on day 0. The samples were coded with randomly selected three-digit numbers, presented individually on white plates, and served to the panelists in a randomized order. The study protocol was approved by the Bioethics Committee of the University of Murcia (approval code: M10/2024/444). All participants were informed about the study objectives and signed an informed consent form.

### 2.8. Statistical Analysis

Differences among GF cookies for all measured parameters, including nutritional and sensory attributes, were evaluated using one-way analysis of variance (ANOVA). A significance level of *p* < 0.05 was applied. Multiple comparisons were assessed using Tukey’s post hoc test, while differences between by-products were evaluated by Student’s *t*-test. All results were expressed as mean ± standard deviation (SD), and analyses were carried out with SPSS Statistics v28.0 software (IBM Corporation, Armonk, NY, USA). Before statistical analysis, sensory data were normalized, and a Principal Component Analysis (PCA) biplot was generated using R software v4.5.0 (R Foundation for Statistical Computing, Vienna, Austria) with the ‘factoextra’ package (v2.5.0) to visualize sample distributions and the contribution of individual sensory attributes to overall variance. In addition, nutritional and physicochemical parameters were analyzed using two-way ANOVA, with by-product treatment (MC and MU) and inclusion level (4% and 8%) as fixed factors. When the Treatment × Inclusion interaction was significant (*p* < 0.05), simple effects were examined using Bonferroni-adjusted pairwise comparisons. The results of these analyses are presented in Table S1.

Multivariate chemometric analyses of metabolomic data were performed on the MetaboAnalyst 6.0 platform (https://www.metaboanalyst.ca). Prior to analysis, abundance values were log-transformed, normalized to the median values of all samples, and auto-scaled (mean-centered and divided by the SD of each variable). Unsupervised hierarchical clustering analysis (HCA) was conducted using heatmaps based on normalized fold-change values, applying Euclidean distances and Ward’s linkage method. This approach allowed evaluation of overall metabolome similarities across experimental groups and identification of potential outliers [25]. PCA was additionally applied to explore the dimensionality and distribution of the metabolomic dataset. The quality of PCA models was assessed using the R^2^ fitting parameter, and statistical significance was confirmed through permutational multivariate analysis of variance (PERMANOVA), with the significance threshold at α = 0.05. To identify metabolites with significant differences between by-products and GF cookies, pairwise Volcano analysis was performed by comparing each treatment against its respective reference sample. Specifically, for by-products, MU was compared to MC, while for GF cookies, each formulation was compared against CC. Volcano analysis combined an unpaired *t*-test (α = 0.05) with a fold-change (FC) cutoff of |±2|, and false discovery rate (FDR) correction was applied for multiple testing. Supervised pairwise orthogonal partial least squares discriminant analysis (OPLS-DA) was further performed to predict differential outcomes based on metabolic profiles. Model quality was evaluated using goodness-of-fit (R^2^Y) and predictive ability (Q^2^), while overfitting was excluded by performing 100-permutation tests (Figure S1). Variable importance in projection (VIP) analysis was conducted to determine the metabolites contributing most strongly to class separation, with VIP scores ≥ 1.0. Differentially accumulated metabolites (DAMs) were identified as features filtered by both Volcano and VIP analyses. DAMs were subsequently subjected to chemical enrichment analysis using ChemRICH [41]. Finally, to test the changes in abundance for each phenolic subclass in response to UAE treatment in the case of mushroom by-products, and the impact of cookie enrichment with mushroom by-products, a class analysis was performed by calculating the cumulative FC of individual metabolites within each phenolic subclass, as applied elsewhere [26].

## 3. Results and Discussion

### 3.1. Chemical Composition

Table 2 summarizes the chemical composition, mineral profile, antioxidant capacity, and TPC of the GF cookies and the mushroom by-products evaluated in this study.

The proximate composition of the mushroom by-products differed significantly between MC and MU (*p* < 0.05). Compared with MC, MU exhibited higher protein content (13.86 vs. 8.79 g/100 g) and ash (13.85 vs. 4.94 g/100 g), while both by-products maintained a low-fat content (<1.1 g/100 g). Regarding dietary fiber, MC was markedly richer in insoluble dietary fiber (IDF), with 49.50 g/100 g, compared to 15.26 g/100 g in MU, representing a difference of more than threefold. Conversely, MU exhibited 13.38 g/100 g of soluble dietary fiber (SDF), approximately twice the amount found in MC (6.26 g/100 g). This shift in dietary fiber is consistent with previous studies reporting that high-intensity ultrasound promotes cell wall disruption, reduces polysaccharide crystallinity, and enhances the solubilization of structural polysaccharides through acoustic cavitation [42]. Similar effects have been reported by Wei et al. [43], who demonstrated that ultrasonic treatment enhanced the SDF content of millet bran by decreasing cellulose rigidity and increasing porosity; similarly, Zhang et al. [44] observed higher SDF yields in UAE of soy-sauce residue due to improved cell-wall disruption; and Vilcapoma et al. [45] reported that cavitation significantly increased SDF in dragon-fruit peel powders by releasing polysaccharides. However, it should be considered that the UAE process also included solvent extraction and filtration steps, which may have influenced the recovery of soluble and insoluble fractions. Therefore, the observed changes in dietary fiber composition are likely the result of the combined effect of ultrasound-induced structural modifications and the extraction procedure, and the individual contribution of each processing step cannot be distinguished under the experimental conditions employed. These compositional changes observed between MC and MU were only partially reflected in the GF cookies.

Regarding fat content, the reformulated cookies generally showed lower values than CC, with the greatest reduction observed in C-4MU, which contained approximately 28% less fat than CC (14.93 vs. 20.84 g/100 g). The two-way ANOVA also showed a significant treatment effect on fat content (*p* = 0.007; Table S1). For dietary fiber, the effects of by-product treatment and inclusion level were interdependent for IDF, SDF, and TDF (Treatment × Inclusion, *p* ≤ 0.021). C-8MC exhibited the highest TDF value (24.65 g/100 g), representing a 15.6% increase relative to CC (21.32 g/100 g), as well as the highest IDF content (20.89 g/100 g). Bonferroni-adjusted comparisons confirmed that, at 8% inclusion, MC and MU differed in IDF, SDF, and TDF; increasing MC inclusion from 4% to 8% also affected all three fiber measures (Table S1). However, despite the clear enhancement of protein and SDF in the MU, protein differences between treatments were significant at 8% inclusion, while the SDF response depended on both treatment and inclusion level (Table S1). This may reflect the contribution of base ingredients such as teff and carob flours, which naturally contain high protein and both soluble and insoluble fiber, thereby moderating the relative contributions of the mushroom by-products [46,47,48]. Nevertheless, all reformulated cookies contained more than 6 g of total dietary fiber per 100 g, meeting the criterion for the “high fiber” nutritional claim established by Regulation (EC) No 1924/2006 [49]. Finally, comparison of CC and COM revealed notable nutritional differences. COM had lower protein (3.53 vs. 5.40 g/100 g) and TDF (6.15 vs. 21.32 g/100 g) contents than CC but higher carbohydrate content (65.36 vs. 35.91 g/100 g) and energy value (462.31 vs. 352.84 kcal/100 g). Because carbohydrates were calculated by difference according to FAO guidelines, the lower carbohydrate content of the developed cookies primarily reflects their higher dietary fiber, moisture, protein, and ash contents. Overall, the experimental cookies contained more protein and dietary fiber than the commercial reference. However, these differences cannot be attributed solely to the incorporation of mushroom by-products because the commercial product differed substantially in its formulation and ingredient composition.

In addition, Table 2 also presents the mineral composition of the GF cookies and mushroom by-products. Details of the other macro- and trace elements analyzed can be found in Table S2. Regarding mushroom by-products, MU showed significantly (*p* < 0.05) higher ash and essential mineral contents, including zinc (Zn), copper (Cu), manganese (Mn), and potassium (K), compared with MC. These differences are consistent with previous studies reporting that high-intensity ultrasound can promote cell wall disruption, thereby facilitating the release and solubilization of minerals associated with the cellular matrix [50]. However, it should also be considered that the extraction protocol included filtration and recovery steps, which may have influenced the retention of mineral-containing fractions. Therefore, the observed mineral composition is likely the result of the combined effects of ultrasound treatment and the extraction process, and the relative contribution of each factor cannot be distinguished under the present experimental conditions. Among the increased elements, Zn, Cu, and Mn play essential protective roles against reactive oxygen species (ROS) due to their function as cofactors of key enzymes such as Cu/Zn-superoxide dismutase (Cu/Zn-SOD) and manganese-dependent SOD (Mn-SOD) [51,52]. These metalloenzymes catalyze the dismutation of superoxide radicals into less reactive molecules, thereby limiting oxidative stress and associated cellular damage. Although MU exhibited higher concentrations of several essential minerals than MC, these differences were only partially reflected in the reformulated cookies. Slight upward trends were observed for K and Mn, with their highest values observed in C-8MU (0.45 g/100 g and 16.52 mg/kg, respectively), indicating a numerical increase associated with higher MU inclusion. In line with these trends, the two-way ANOVA detected no significant treatment or inclusion effects for the minerals, although the Treatment × Inclusion interaction was significant for sulfur (*p* = 0.046); no Bonferroni-adjusted simple effects were significant (Table S1). Moreover, in all the reformulated cookies, iron (Fe) emerged as the most nutritionally relevant mineral with values ranging from 44.51 mg/kg (C-8MC) to 56.73 mg/kg (CC). According to Regulation (EU) No 1169/2011, the Nutrient Reference Value for iron is 14 mg/day, and a significant amount for a solid food corresponds to 15% of this value per 100 g, equivalent to 2.1 mg of iron per 100 g [53]. The lowest iron concentration measured among the reformulated cookies was 44.51 mg/kg, corresponding to 4.45 mg/100 g and therefore exceeding this threshold. Accordingly, all reformulated cookies meet the criterion for the “source of iron” nutritional claim. Conversely, COM exhibited substantially lower levels of K, Fe, Mn, and Zn, while containing higher sodium (Na) due to its salt content. This mineral profile is consistent with the use of refined gluten-free flours and starches, which generally contain lower concentrations of macro- and microminerals than whole-grain ingredients [54]. This is particularly relevant for celiac patients, who frequently present Fe and Zn deficiencies due to malabsorption and dependence on refined GF products [55]. These differences likely reflect the distinct ingredient composition of commercial and experimental cookies.

The impact of incorporating mushroom by-products into GF cookies on antioxidant capacity and phenolic content was determined using colorimetric assays (FRAP, ABTS, DPPH, and TPC). MU exhibited significantly higher TPC and antioxidant capacity than MC, indicating differences in the antioxidant-related properties of the materials obtained through the two processing approaches. This behavior aligns with the ability of high-intensity ultrasound to break down fungal cell walls and liberate bound phenolic compounds [56]. Optimized UAE has been reported to significantly enhance TPC, as well as DPPH and ABTS activities in mushroom matrices [57]. Similarly, Machado-Carvalho et al. [58] reported that UAE applied to *Inonotus hispidus* maximized phenolic compounds recovery, resulting in marked increases in FRAP and DPPH antioxidant activities. Regarding the reformulated GF cookies, increases in FRAP and ABTS were observed in C-4MU and C-8MU, consistent with the higher antioxidant capacity measured in MU. However, these differences were not statistically significant among the experimental formulations, and DPPH values were also comparable across formulations. This pattern was consistent with the two-way ANOVA, which found no significant treatment, inclusion, or Treatment × Inclusion effects for FRAP, ABTS, or DPPH (Table S2). TPC was slightly lower in MU-fortified cookies, with C-4MU showing a significantly lower value than CC. The two-way ANOVA also showed a treatment effect on TPC (*p* < 0.001), but no significant inclusion effect or Treatment × Inclusion interaction (Table S2). This apparent discrepancy is likely the result of several concurrent factors. Interactions between phenolic compounds and macromolecules such as proteins, starch, and dietary fiber may reduce the extractability of certain phenolics during sample preparation for spectrophotometric assays [59,60]. In addition, phenolic compounds released during UAE may change during baking, including thermal degradation or oxidation [61,62,63]. Furthermore, the extraction and recovery steps involved in obtaining MU could also have contributed to differences in the phenolic fractions available for analysis. Therefore, spectrophotometric assays estimate antioxidant capacity as an overall measure of the extracted fraction and may not fully reflect qualitative changes in phenolic composition within complex food matrices. Importantly, TPC provides an estimate of total phenolic compounds detected under the assay conditions and does not necessarily reflect the concentration or activity of individual phenolic compounds. Accordingly, the lower TPC observed in C-4MU did not necessarily result in lower antioxidant capacity, as FRAP, ABTS, and DPPH remained statistically comparable to CC. Nevertheless, all experimentally formulated GF cookies had higher measured values compared with COM: approximately 185, 152, 10, and 377% higher levels in FRAP, ABTS, DPPH, and TPC, respectively. Overall, these results indicate that the incorporation of mushroom by-products did not consistently increase the overall antioxidant capacity of cookies compared with the control formulation, although the reformulated cookies showed higher measured antioxidant-related parameters than the commercial GF cookie. However, all these comparisons with COM should be interpreted with caution, as the commercial product differs considerably in formulation and ingredient composition. Therefore, the observed differences can be attributed not only to the incorporation of mushroom by-products but also to differences in the overall recipe.

### 3.2. Physicochemical Properties

The physicochemical profile of the different GF cookies was characterized by colorimetry (CIEL*a*b*) and acidity analysis, as presented in Table 3. In terms of color measurements, the incorporation of mushroom by-products (MC and MU) into GF cookies led to modest color shifts compared to CC. The L* parameter (lightness) increased significantly in all enriched formulations, reaching the highest values in MU-enriched cookies (30.72 and 31.38 in C-4MU and C-8MU, respectively), indicating a lighter appearance. This difference may be mainly related to the formulation and the thermal processes occurring during baking. In particular, the high baking temperature may have influenced the development of browning reactions, while the incorporation of the mushroom extract in MU-enriched cookies may have modified the composition and distribution of compounds involved in these reactions, contributing to their lighter appearance [64]. In the two-way ANOVA, L* was affected by treatment (*p* < 0.001) and inclusion level (*p* = 0.018), with no significant Treatment × Inclusion interaction (Table S2). Additionally, all mushroom-enriched GF cookies showed a simultaneous increase in chroma (*C*) and hue (*h*) values, indicating a shift toward a more intense and yellowish color, consistent with the increased b* value observed in MU-enriched samples (9.65 and 10.62 for C-4MU and C-8MU, respectively). The two-way ANOVA showed no significant effects for chroma, whereas hue and b* were affected by treatment and inclusion level (Table S2). Color differences relative to the control, quantified using ΔE*, ranged from 0.88 (C-4MC) to 3.49 (C-8MU). Visual perception studies commonly consider ΔE* values above 3 units as detectable by the human eye [65]. Consistent with this, samples containing low mushroom powder concentrations showed minimal perceptible changes, while higher concentrations, particularly with UAE-treated powder, led to more noticeable alterations in lightness and chromaticity. These observations are consistent with previous reports on bakery products enriched with mushroom powder, such as those by Salehi et al. [66], where color changes increased proportionally with the concentration of mushroom by-products. Collectively, these findings demonstrate that incorporating mushroom by-products modulates cookie color in a dose- and treatment-dependent manner. The lack of significant Treatment × Inclusion interactions indicates that the effects on L*, *h*, and b* were not specific to a particular combination of treatment and inclusion level. In contrast, COM was markedly lighter and more saturated than CC, yielding a huge overall color difference (∆E* = 46.20). These values show that the experimental formulation containing carob flour had a fundamentally darker, less saturated, reddish-brown appearance compared with the commercial product, which includes refined yellowish flours [67,68].

In addition, Table 3 also shows the results related to the pH and total titratable acidity (TTA) of the different GF cookies. All samples presented slightly acidic pH values, ranging from 5.46 (CC) to 5.95 (C-8MC). Among the reformulated cookies, pH was affected by inclusion level and by the Treatment × Inclusion interaction. Bonferroni-adjusted comparisons showed differences between MC and MU at 8% inclusion (*p* = 0.014), and between the two inclusion levels for MC (*p* < 0.001; Table S2). Moreover, TTA values differed significantly (*p* < 0.05). Among the reformulated cookies, C-8MU exhibited the highest acidity (9.42 mL NaOH/10 g), followed by CC (9.10 mL NaOH/10 g). The two-way ANOVA showed a Treatment × Inclusion interaction for TTA (*p* < 0.001). At 8% inclusion, MC and MU differed significantly, and increasing inclusion from 4% to 8% significantly affected TTA for both MC and MU (all *p* < 0.001; Table S2). These differences in TTA indicate changes in the titratable acid content of the samples, even though their pH values were within a relatively narrow range. This trend can be explained by the composition of teff and carob flours, which are naturally rich in fiber and phenolic compounds, such as gallic and chlorogenic acids, which contribute significantly to TTA [46,47]. Regarding the effect of incorporating mushroom by-products, the data revealed contrasting effects. At the 4% inclusion level, both MC and MU produced a slight decrease in acidity compared with CC, which can be attributed to the partial substitution of the teff–carob matrix with mushroom-derived material. At the 8% inclusion level, the effects of the two by-products diverged: C-8MC showed lower TTA than CC, consistent with a dilution effect, whereas C-8MU exhibited a pronounced increase and had the highest TTA among the formulations. This contrasting effect may be linked to the structural disruption promoted by ultrasound processing, which is known to enhance the release of intracellular components such as organic acids (e.g., citric, malic), phenolic acids (gallic, chlorogenic), and other polyphenols [69,70]. Consequently, at elevated concentrations, MU may contribute additional extractable organic acids or phenolic compounds to the dough, while MC ingredients predominantly exert a dilution-driven effect. Finally, COM exhibited the lowest acidity (2.53 mL NaOH/10 g), underscoring the difference between refined ingredients and those naturally rich in bioactive compounds.

### 3.3. Untargeted Phenolic Profiling of Mushroom By-Products and Gluten-Free Cookies

An untargeted metabolomics approach was employed to characterize the phenolic profiles of oyster mushroom by-products (MC and MU) and the different GF cookie formulations. Using the Phenol-Explorer database, 291 chemical entities were putatively annotated (Table S3). Flavonoids were the most prevalent class of polyphenols, comprising 145 annotated metabolites, predominantly flavonols (36) and anthocyanins (35). The flavonols mainly consisted of kaempferol, quercetin, and myricetin conjugates, often exhibiting complex glycosylation patterns. The anthocyanins were predominantly annotated as glycosylated derivatives of cyanidin, delphinidin, and malvidin. Other well-represented flavonoid subclasses included isoflavonoids (29), flavanols (19), and flavanones (13). Phenolic acids were the second-most prevalent class, comprising 57 metabolites, primarily hydroxycinnamic acids (34), including caffeic, ferulic, and p-coumaric acids, followed by hydroxybenzoic acids (11) and hydroxyphenylpropanoic acids (8). The category of other polyphenols comprised 53 metabolites, with alkylphenols (10) and tyrosols (10) as the most frequent subgroups. Lignans (22) and stilbenes (12) were also annotated but in smaller numbers. Given the complexity of the dataset and the broad range of chemical families represented, multivariate statistical methods were used to interpret differences among treatments and evaluate the effects of mushroom by-product incorporation on the phenolic profiles of the GF cookies.

#### 3.3.1. Metabolomic Profiling and Chemometric Analysis of Mushroom By-Products

**Figure 2:**
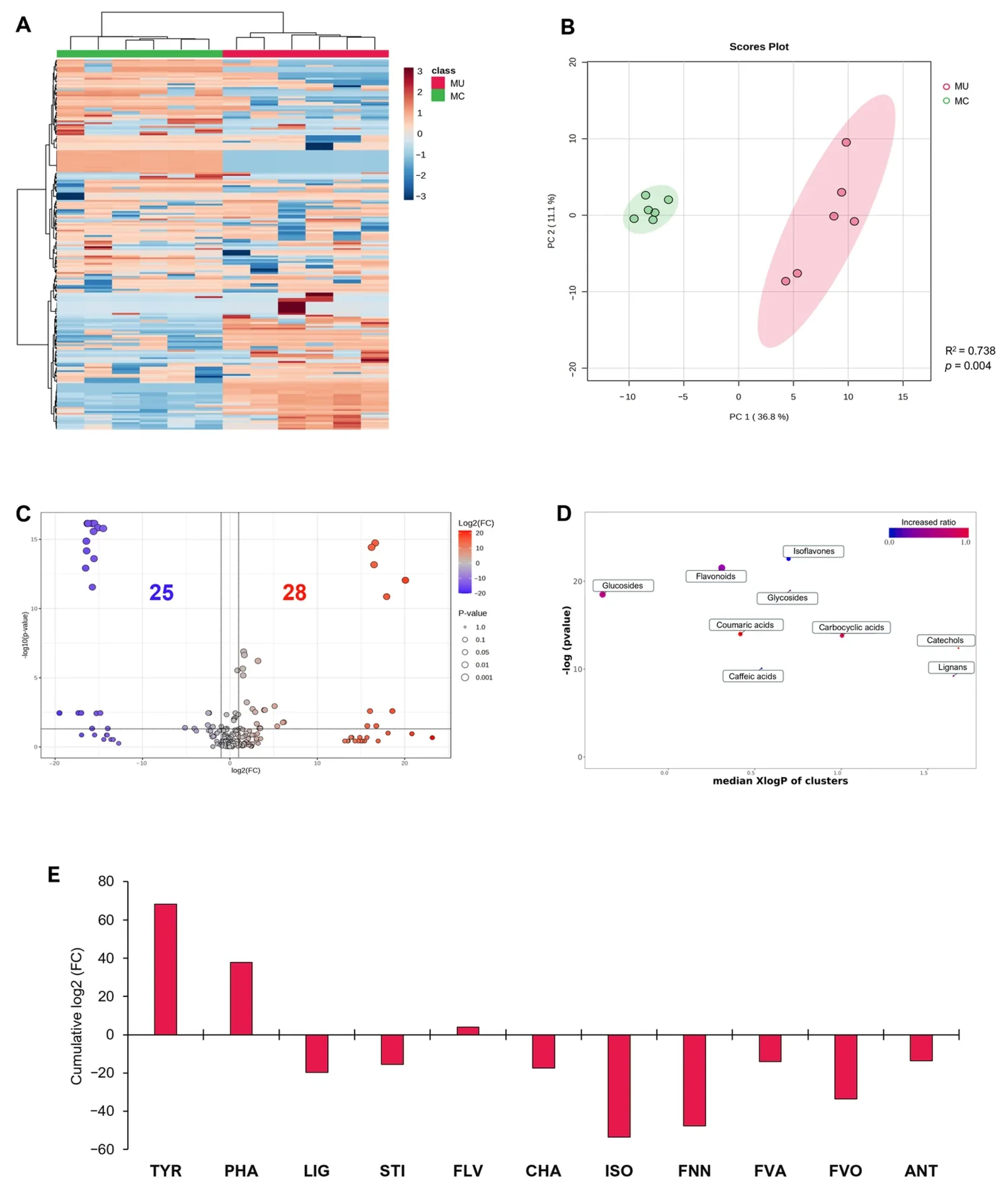
Chemometric analysis of the untargeted UHPLC-QTOF-HRMS metabolomic profiling of mushroom by-products. (**A**) Hierarchical cluster analysis (HCA) based on a fold change heatmap (Euclidean distance, Ward’s clustering algorithm). The color scale represents the FC values of each detected feature relative to the overall median across treatments. (**B**) Principal component analysis (PCA), R^2^ = 0.738, PERMANOVA *p* = 0.004. (**C**) Volcano plot (log_2_(FC) ≥ |±2|; *p* < 0.05) of the differential metabolites derived from MU compared to MC. (**D**) Chemical similarity enrichment analysis (ChemRICH) derived from DAMs of MU compared to MC (VIP score > 1). Each point represents a group of significantly altered compounds; point size reflects the number of metabolites, and the color indicates the accumulation trend compared to the control. Compound classes are ranked on the *y*-axis according to significance, and polarity increases along the *x*-axis. (**E**) Class analysis based on the cumulative individual log_2_(FC) values of the metabolites included within each phenolic subfamily, according to Phenol-Explorer classification, setting MC as the reference control. Abbreviations: MC: control mushroom by-products; MU: UAE-treated mushroom by-products; PC1: first principal component; PC2: second principal component; TYR: tyrosol and other phenolics; PHA: phenolic acids; LIG: lignans; STI: stilbenes; FLV: flavones; CHA: chalcones; ISO: isoflavones; FNN: flavanones; FVA: flavanols; FVO: flavonols; ANT: anthocyanins.

To explore the global impact of UAE treatment on the metabolomic profile of mushroom by-products, multivariate chemometric approaches were applied (Figure 2). Unsupervised analyses, including hierarchical cluster analysis (HCA) and principal component analysis (PCA), were first conducted to explore the inherent similarities and differences between MC and MU. HCA (Figure 2A) revealed a clear separation between the two samples, indicating distinct phenolic profiles and highlighting the influence of UAE on the phenolic composition of mushroom by-products. These results were consistent with those obtained by PCA (Figure 2B), where the score plot showed a clear separation between MC and MU. The clustering observed in the model was statistically supported by PERMANOVA (R^2^ = 0.738; *p* = 0.004), confirming significant differences among the groups.

To further explore the impact of UAE, supervised modeling of the metabolomic profiles of mushroom by-products was performed. A Volcano analysis (log_2_(FC) ≥ |±2|, *t*-test *p* < 0.05) was conducted to identify those features with statistically significant differences in their abundance between samples, using MC as the reference condition. A total of 53 metabolites were filtered by Volcano’s parameters, suggesting a pronounced modulation of the phenolic composition induced by UAE (Figure 2C). An orthogonal projection to latent structures discriminant analysis (OPLS-DA) was performed in parallel, yielding robust performance metrics (R^2^Y = 0.975 and Q^2^ = 0.925), which confirmed the clear separation between groups according to their phenolic profile. The VIP markers associated with this model were combined with those provided by Volcano analysis to obtain the differentially accumulated metabolites (DAMs), resulting in a total of 53 DAMs (Table S5). Those DAMs were further subjected to ChemRICH analysis, revealing significant differences in the accumulation of phenolic clusters of MU compared to MC (Table S6). The enrichment plot (Figure 2D) revealed substantial alterations in several phenolic subclasses. Coumaric acids, carbocyclic acids, and catechols were consistently enriched, indicating an increased accumulation of phenolic acids and low-molecular-weight (LMW) phenolics. Glucosides also exhibited substantial changes, indicating a balanced redistribution of conjugated phenolics. In contrast, flavonoids and isoflavones largely decreased, while glycosides and lignans showed moderate alterations. Class analysis based on the cumulative individual log_2_(FC) values of the metabolites supported these patterns (Figure 2E), where MU exhibited significantly higher levels of phenolic acids and tyrosols, and other phenolics, whereas isoflavones, flavanones, and flavonols showed the greatest reductions compared with MC. Moderate decreases were also observed for flavanols, lignans, anthocyanins, and stilbenes, while flavones remained relatively unchanged.

The integration of ChemRICH and class analysis confirms the selective effect of UAE: rather than uniformly increasing total phenolic content, UAE redistributes phenolic subclasses, favoring the extraction of simple, easily extractable molecules while reducing the extraction of complex flavonoids. This behavior may be explained by the mechanism of acoustic cavitation. The collapse of microbubbles generates microjets, shear forces, and localized thermal and pressure gradients. These phenomena subsequently fragment cell walls and induce sonoporation, thereby increasing solid–liquid contact and promoting the solubilization and release of phenolics previously bound to the matrix [50,71]. Simultaneously, these conditions can destabilize complex polyphenols (flavanols, isoflavones, anthocyanins), leading to deconjugation, partial oxidation, depolymerization, or isomerization. As a result, complex phenolic compounds decrease while simple phenolic clusters accumulate [50,72]. Therefore, the UAE not only enhances extractability but also shifts the phenolic profile toward compounds contributing most to the observed antioxidant capacity (Table 2). Similar modulatory effects of UAE have been reported in different plant matrices. Casas-Rodriguez et al. [73] reported that UAE applied to pineapple peel significantly increased hydroxycinnamic acids, particularly caffeoylquinic derivatives, accompanied by a clear rise in LMW phenolics due to cavitation-induced cell disruption. Moreover, another study evaluated UAE-treated grape pomace, which showed an increase in the release of free phenolics by liberating cell-wall-bound compounds [74]. In sunflower seed shells, Keskin et al. [75] found that UAE markedly improved the extractability and bioaccessibility of hydroxycinnamic acids, reinforcing the hypothesis that ultrasonic cavitation favors the accumulation of simple phenolics over structurally complex flavonoids. Overall, the UAE acts as a selective modulator of phenolic composition, promoting the extraction of compounds with greater bioaccessibility and functional relevance while reducing that of complex structures prone to degradation.

#### 3.3.2. Metabolomic Profiling of Gluten-Free Cookies and Chemometric Analysis

**Figure 3:**
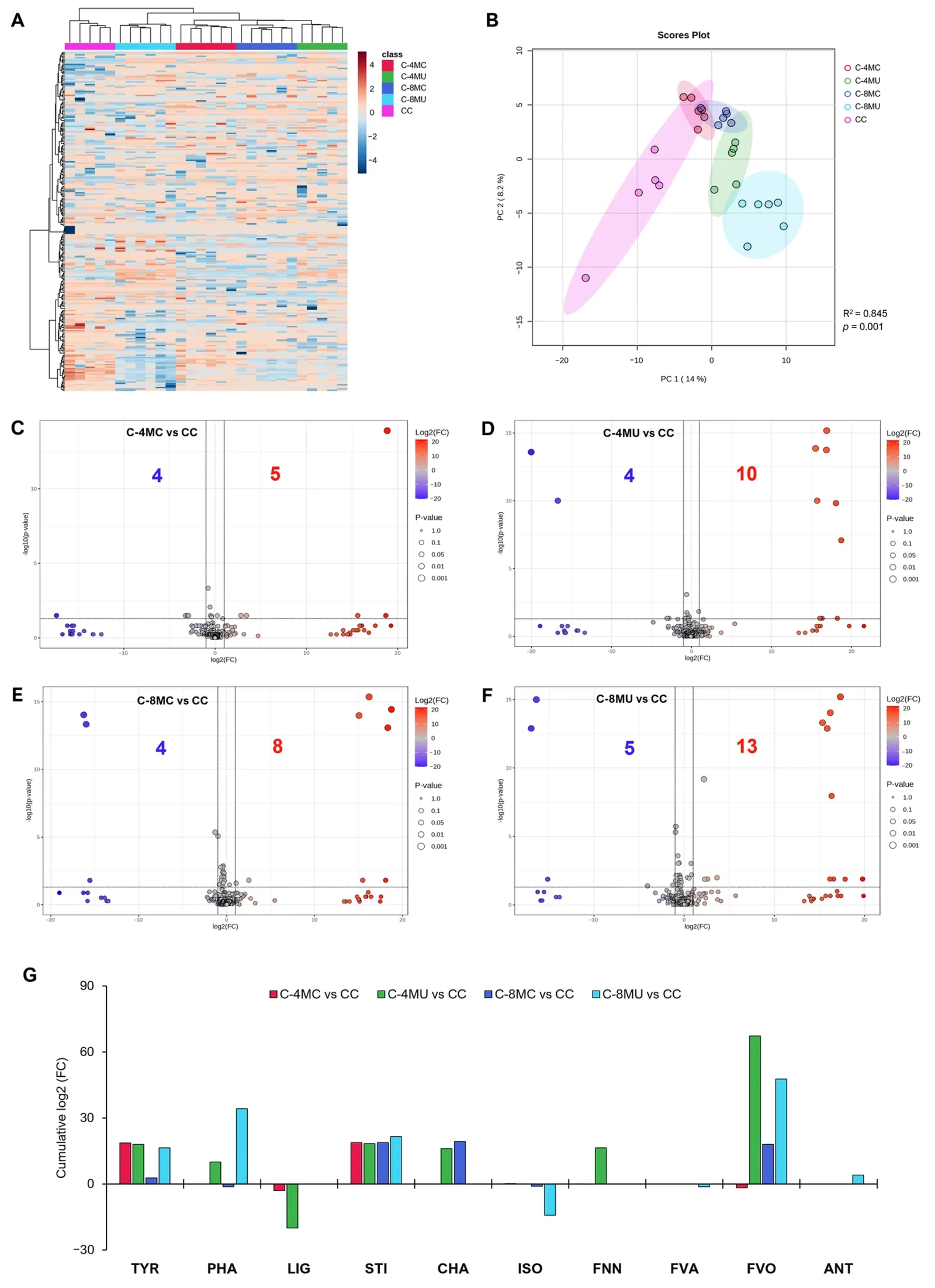
Chemometric analysis of the untargeted UHPLC-QTOF-HRMS metabolomic profiling of gluten-free cookies. (**A**) Hierarchical cluster analysis (HCA) based on a fold-change heatmap (Euclidean distance, Ward’s clustering algorithm). The color scale represents the FC values of each detected feature relative to the overall median across treatments. (**B**) Principal component analysis (PCA), R^2^ = 0.845, PERMANOVA *p* = 0.001. (**C**–**F**). Volcano plots (log2(FC) ≥ |±2|; *p* < 0.05) of the differential metabolites derived from C-4MC (**C**), C-4MU (**D**), C-8MC (**E**), and C-8MU (**F**) compared with CC. In all plots, colors indicate the direction and significance of metabolite changes in mushroom-enriched cookies compared with CC, distinguishing significantly upregulated and downregulated metabolites from non-significant features. Dashed lines indicate the significance thresholds (*p* < 0.05 and FDR-adjusted *p*-value). (**G**) Class analysis based on the cumulative individual log2(FC) values of the metabolites included within each phenolic subfamily, according to Phenol-Explorer classification, setting CC as the reference control. Abbreviations: CC: gluten-free control cookie; C-4MC: gluten-free cookie with 4% MC; C-4MU: gluten-free cookie with 4% MU; C-8MC: gluten-free cookie with 8% MC; C-8MU: gluten-free cookie with 8% MU; COM: commercial gluten-free cookie; PC1: first principal component; PC2: second principal component; TYR: tyrosol and other phenolics; PHA: phenolic acids; LIG: lignans; STI: stilbenes; FLV: flavones; CHA: chalcones; ISO: isoflavones; FNN: flavanones; FVA: flavanols; FVO: flavonols; ANT: anthocyanins.

In parallel, multivariate analysis was employed to assess the metabolomic profiles of the six cookie formulations. Unsupervised chemometrics, including HCA and PCA, revealed a marked difference between the COM sample and all cookie formulations, masking the effects of the mushroom by-product reformulations and the effect attributed to UAE (Figure S2A,B). For this reason, the chemometric analysis was performed with only the five GF cookie formulations. Figure 3 presents the results of the multivariate analysis on the untargeted phenolic profile of GF cookie formulations. The five experimental formulations converged into two main clusters: one containing only CC, and another including all formulations containing mushroom by-products (Figure 3A). Furthermore, these trends were confirmed by the PCA (R_2_ = 0.845, PERMANOVA *p* = 0.001) (Figure 3B). The formulations with treated by-products (MU) exhibited a greater distance from the CC, while those containing MC were closer to the control formulation, with PC1 explaining 14% of total variation.

Supervised analyses on the metabolic profile of the formulations were performed, setting CC as the reference treatment. Consequently, Volcano analysis (considering log2(FC) ≥ |±2|; *p* < 0.05) (Figure 3C–F) and OPLS-DA modeling (R^2^Y ≥ 0.933 and Q^2^ ≥ 0.739) were performed to identify the DAMs. (Table S6 and Table S7, respectively). These analyses revealed a progressive increase in the number of DAMs with increasing inclusion level and UAE treatment. In particular, the number of upregulated DAMs increased from 5 in C-4MC to 13 in C-8MU compared to CC. Due to the limited number of DAMs identified for each comparison (9, 14, 12, and 18 for C-4MC, C-4MU, C-8MC, and C-8MU vs. CC, respectively), ChemRICH analysis could not be applied, as the small dataset is insufficient to generate statistically meaningful metabolite clusters. However, class analysis (Figure 3G) provided an overview of the effect on major phenolic families among enriched cookies compared to CC. The incorporation of mushroom by-products modified the phenolic composition of the cookies, with more pronounced changes observed in formulations containing MU. C-4MU and C-8MU exhibited a greater enrichment of flavonols and phenolic acids, together with positive changes in tyrosols and other phenolics and stilbenes. These results are consistent with those of raw mushroom by-products, where UAE increased the bioaccessibility of phenolic acids by disrupting fungal cell walls. In contrast, cookies containing MC showed only moderate or no enrichment of most phenolic classes. This behavior is consistent with the limited availability of phenolic compounds covalently bound to structural polymers in MC [76].

However, these changes were less pronounced than those observed in the raw mushroom by-products, suggesting that dilution within the complex food matrix, interactions between phenolic compounds and macromolecules such as proteins, starch, and dietary fiber, together with thermal processing during baking, may have reduced the extractability and stability of individual phenolic compounds [77,78]. Consequently, although conventional antioxidant assays detected only limited differences among cookie formulations, the untargeted metabolomic approach revealed qualitative modifications in phenolic composition that were not fully captured by global spectrophotometric measurements. This indicates that UAE may primarily influence the distribution of individual phenolic subclasses rather than producing a proportional increase in the overall antioxidant capacity of the final product.

### 3.4. Sensory Evaluation

**Figure 4:**
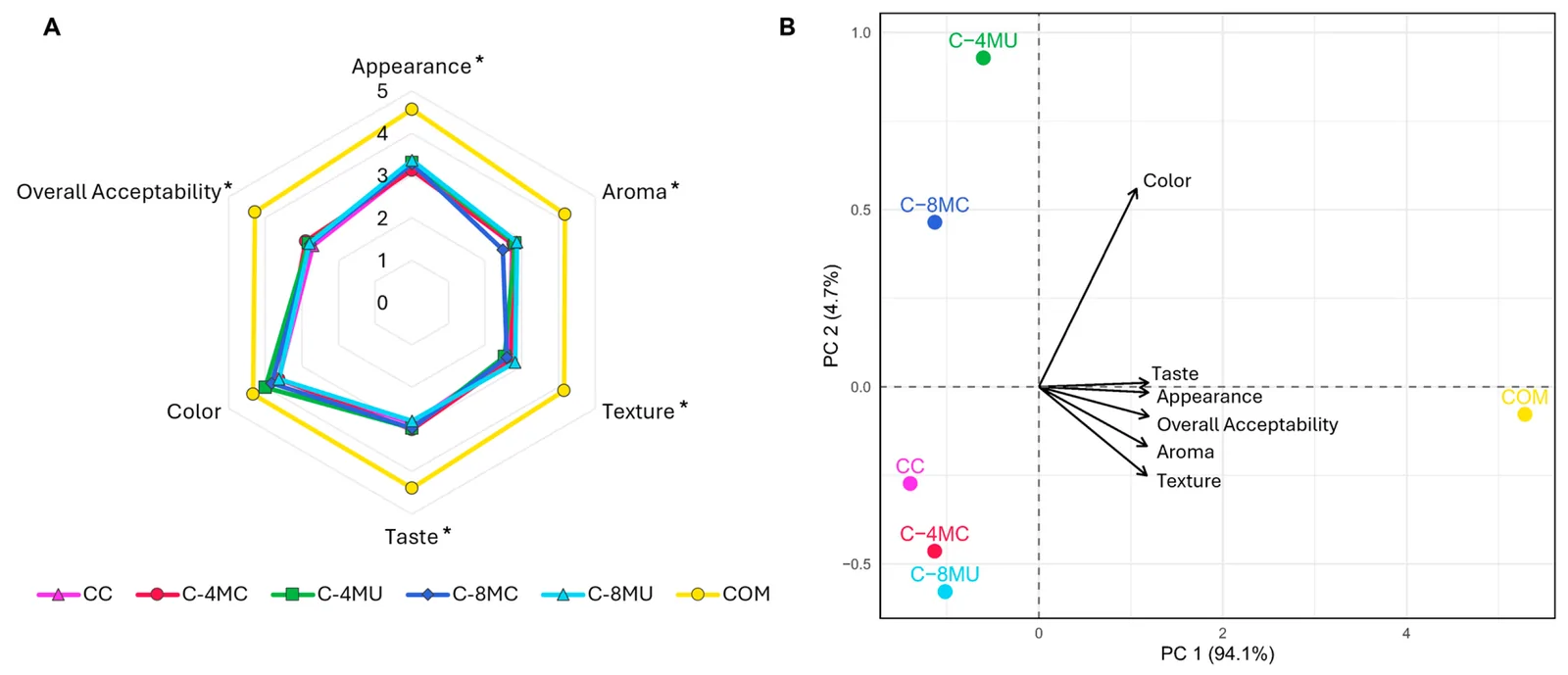
Sensory analysis of gluten-free cookie formulations. (**A**) Radar plot of the sensory attributes for the different formulations. (**B**) Principal component analysis (PCA) biplot illustrating the distribution of the gluten-free cookies according to their sensory profiles and the contribution of each attribute to sample differentiation (* *p* < 0.05). Dashed lines indicate the zero values of PC1 and PC2. Abbreviations: CC: gluten-free control cookie; C-4MC: gluten-free cookie with 4% MC; C-4MU: gluten-free cookie with 4% MU; C-8MC: gluten-free cookie with 8% MC; C-8MU: gluten-free cookie with 8% MU; COM: commercial gluten-free cookie.

A consumer panel evaluated the sensory characteristics of the different GF cookie formulations, and the results for each attribute are presented in Figure 4. The radar plot (Figure 4A) shows that formulations enriched with mushroom by-products, regardless of treatment or inclusion level, showed sensory scores generally comparable to those of CC, particularly in terms of texture and taste. Moreover, overall acceptability remained stable even at higher inclusion levels (8%) and under ultrasound treatment, suggesting that these formulations did not produce substantial changes in the sensory scores under the conditions evaluated. These findings are consistent with the nutritional and physicochemical characterization, which showed relatively small differences in the overall composition of the experimental formulations. The moderate changes in dietary fiber content, together with the similar moisture and protein contents, may have contributed to preserving key sensory attributes such as texture and overall acceptability. In addition, these results are consistent with previous findings by Shams et al. [79], who reported that mushroom powder can be added up to approximately 10% without negatively affecting sensory scores, while higher levels tend to reduce scores due to changes in color, texture, and taste. In contrast, COM achieved the highest scores across all attributes (*p* < 0.05). Its formulation includes emulsifiers (lecithin), hydrocolloids (guar gum, xanthan gum), and flavoring agents, which have been shown to enhance texture, moisture retention, and taste in bakery products, potentially contributing to its higher sensory scores [80,81,82]. However, the commercial product had a substantially different formulation and ingredient composition from the experimental cookies, and its higher sensory scores should therefore be interpreted in the context of these compositional differences.

For a deeper understanding of how sensory attributes contributed to the differentiation among formulations, a PCA plot was generated based on the sensory scores, as shown in Figure 4B. PCA explained 94.1% of the variance on PC1 and 4.7% on PC2. The COM formulation was clearly separated from the others along PC1, clustering with positive attributes such as aroma, appearance, and overall acceptability, reflecting its higher scores for these attributes. In contrast, all reformulated GF cookies were positioned on the negative side of PC1, indicating lower association with these attributes. CC and C-4MC were positioned close to each other, indicating similar sensory characteristics, while C-8MU remained near CC with minimal variation. However, C-4MU and C-8MC showed some displacement along PC2, suggesting slight differences mainly related to color. These patterns indicate that incorporating mushroom by-products into GF cookie formulations did not substantially alter the sensory scores compared to the control under the conditions evaluated, supporting the potential use of these functional ingredients in clean-label applications [83,84]. Similar results have been reported for GF bakery products enriched with plant-derived ingredients, such as *Moringa oleifera* [85], *Musa anamalu* [86], and *Daucus carota* [87], as well as microalgae like *Arthrospira platensis* [9]. Considering the nutritional improvements, the sensory evaluation, and the phenolic enrichment revealed by metabolomics, C-8MC and C-8MU showed favorable combinations of the measured nutritional, sensory, and metabolomic characteristics and should be further investigated. C-8MC achieved the highest dietary fiber content among all reformulated cookies, whereas C-8MU displayed the most enriched mineral and phenolic profile, reflecting the functional potential unlocked by UAE. Although these findings provide valuable preliminary evidence from a limited consumer panel, validation in larger consumer populations would further strengthen the robustness and generalizability of the sensory outcomes.

## 4. Conclusions

This study elucidates the potential of *Pleurotus ostreatus* mushroom by-products as sustainable and nutritionally valuable ingredients for the development of nutritionally enhanced GF cookies. Moreover, the material obtained through the UAE process (including UAE-treated material that underwent solvent extraction and fractionation) showed higher protein, soluble dietary fiber, and concentrations of several essential minerals (Zn, Cu, and Mn) than the whole mushroom by-product powder, alongside differences in phenolic composition. Rather than uniformly increasing all phenolic compounds, the UAE process promoted a redistribution of phenolic subclasses, favoring phenolic acids and other low-molecular-weight phenolics of interest due to their reported bioactive properties.

The incorporation of these by-products into gluten-free cookies resulted in formulations with an enhanced nutritional profile compared with a commercial GF reference. The reformulated cookies showed higher protein and total dietary fiber contents, fulfilling the “high fiber” nutritional claim, together with a more balanced mineral profile, particularly regarding iron, which is especially relevant considering the high prevalence of iron deficiency among individuals with celiac disease. These nutritional improvements were achieved while maintaining sensory characteristics comparable to those of the control formulation. The compositional differences between MC and MU were not consistently reflected in the nutritional and antioxidant properties of the reformulated cookies. Although some parameters differed between formulations, significant changes were not observed for all the variables evaluated. Furthermore, comparisons with the commercial gluten-free cookie should be interpreted with caution because of differences in ingredient composition and formulation.

Although all the UAE process did not consistently increase the overall antioxidant capacity of the baked cookies, untargeted metabolomic analysis revealed qualitative modifications in their phenolic composition that were not fully captured by conventional spectrophotometric assays, highlighting the complementary value of both analytical approaches for evaluating the effects of the complete extraction process on the phenolic composition of reformulated cookies. Among the reformulated cookies, C-8MC showed a favorable combination of nutritional and sensory characteristics, particularly its high dietary fiber content and favorable sensory scores, and should be further investigated. In contrast, C-8MU exhibited the most differentiated phenolic profile and higher levels of potassium and manganese, indicating a distinct phenolic and mineral profile that should also be further investigated.

Overall, combining agro-industrial by-product valorization with UAE process represents a promising strategy for developing clean-label GF bakery products with improved nutritional quality, while supporting the principles of the circular economy. However, potential health effects, bioavailability of the identified compounds, industrial applicability, and commercial feasibility were not assessed in the present study and should be addressed in future research. Furthermore, larger and more representative consumer studies are needed to further evaluate the sensory performance of the formulations and their potential for commercial development. Future research should focus on assessing bioaccessibility and bioavailability, evaluating biological effects, validating product stability during storage, and assessing processing at pilot or industrial scale to determine commercial feasibility.

## Figures and Tables

**Table 1:** Formulation of gluten-free cookies.

Ingredients	CC	C-4MC	C-4MU	C-8MC	C-8MU
Teff flour (g)	87.5	87.5	87.5	87.5	87.5
Carob flour (g)	87.5	87.5	87.5	87.5	87.5
Sugar (g)	65	65	65	65	65
Dark chocolate (g)	40	40	40	40	40
Olive oil (g)	50	50	50	50	50
Egg (unit)	1	1	1	1	1
Milk (mL)	52	52	52	52	52
MC (g)	-	7	-	14	-
MU (g)	-	-	7	-	14

Abbreviations: MC: control mushroom by-products; MU: UAE-treated mushroom by-products; CC: gluten-free control cookie; C-4MC: gluten-free cookie with 4% MC; C-4MU: gluten-free cookie with 4% MU; C-8MC: gluten-free cookie with 8% MC; C-8MU: gluten-free cookie with 8% MU.

**Table 2:** Proximate composition, trace elements, antioxidant activity, and total phenolic content (TPC) of mushroom by-products and gluten-free cookies.

	Mushroom By-Products		Gluten-Free Cookies					
	MC	MU	CC	C-4MC	C-4MU	C-8MC	C-8MU	COM
Proximate composition								
Energy content (kcal/100 g)	115.95 ± 10.19 b	178.32 ± 7.91 a	352.84 ± 7.31 bc	360.24 ± 2.65 b	320.28 ± 1.19 d	331.75 ± 14.20 cd	321.98 ± 10.36 d	462.31 ± 6.26 a
Fat (g/100 g)	0.75 ± 0.73 a	1.04 ± 0.28 a	20.84 ± 0.47 a	20.56 ± 0.63 a	14.93 ± 0.26 b	18.63 ± 2.14 ab	17.14 ± 2.60 ab	20.75 ± 1.76 a
Protein (g/100 g)	8.79 ± 0.34 b	13.86 ± 0.13 a	5.40 ± 0.45 a	5.16 ± 0.19 a	5.00 ± 0.08 a	4.84 ± 0.28 a	5.33 ± 0.09 a	3.53 ± 0.14 b
Moisture (g/100 g)	11.85 ± 0.06 b	14.93 ± 0.68 a	15.23 ± 0.82 b	12.63 ± 0.50 c	14.75 ± 0.54 b	14.37 ± 0.73 bc	17.64 ± 1.05 a	3.47 ± 0.78 d
Ash (g/100 g)	4.94 ± 0.029 b	13.85 ± 2.83 a	1.29 ± 0.14 b	1.46 ± 0.22 ab	1.61 ± 0.12 ab	1.33 ± 0.06 b	1.86 ± 0.10 a	0.74 ± 0.21 c
IDF (g/100 g)	49.50 ± 1.48 a	15.26 ± 1.94 b	18.05 ± 0.73 b	18.25 ± 0.54 b	18.61 ± 0.62 b	20.89 ± 0.59 a	18.09 ± 0.60 b	4.61 ± 0.35 c
SDF (g/100 g)	6.26 ± 0.97 b	13.38 ± 0.59 a	3.27 ± 0.38 a	3.30 ± 0.16 a	3.61 ± 0.13 a	3.76 ± 0.14 a	3.33 ± 0.37 a	1.55 ± 0.04 b
TDF (g/100 g)	55.76 ± 2.33 a	28.64 ± 1.65 b	21.32 ± 1.11 b	21.55 ± 0.39 b	22.23 ± 0.49 b	24.65 ± 0.45 a	21.42 ± 0.97 b	6.15 ± 0.31 c
Carbohydrates (g/100 g)	18.70 ± 2.33 b	28.69 ± 1.08 a	35.91 ± 1.22 b	38.64 ± 1.24 b	41.49 ± 0.22 b	36.19 ± 1.76 b	36.60 ± 4.05 b	65.36 ± 2.74 a
Essential Minerals								
Ca (g/100 g)	0.04± 0.00 b	0.06 ± 0.00 a	0.10 ± 0.00 a	0.09 ± 0.01 a	0.09 ± 0.00 a	0.08 ± 0.01 a	0.09 ± 0.00 a	0.01 ± 0.00 b
K (g/100 g)	2.80 ± 0.04 b	5.49 ± 0.06 a	0.34 ± 0.00 a	0.35 ± 0.07 a	0.36 ± 0.02 a	0.36 ± 0.01 a	0.45 ± 0.01 a	0.12 ± 0.00 b
Mg (g/100 g)	0.11 ± 0.00 b	0.23 ± 0.00 a	0.06 ± 0.00 a	0.05 ± 0.01 a	0.05 ± 0.00 a	0.05 ± 0.00 a	0.06 ± 0.00 a	0.03 ± 0.00 b
Na (g/100 g)	0.03 ± 0.00 b	0.09 ± 0.00 a	0.03 ± 0.00 b	0.03 ± 0.00 b	0.02 ± 0.00 b	0.03 ± 0.00 b	0.03 ± 0.00 b	0.18 ± 0.06 a
P (g/100 g)	0.18 ± 0.00 b	0.33 ± 0.01 a	0.18 ± 0.00 a	0.17 ± 0.02 a	0.16 ± 0.00 a	0.15 ± 0.00 ab	0.17 ± 0.01 a	0.11 ± 0.01 b
S (g/100 g)	0.13 ± 0.00 b	0.20 ± 0.00 a	0.09 ± 0.00 a	0.09 ± 0.01 a	0.08 ± 0.00 a	0.08 ± 0.00 a	0.09 ± 0.00 a	0.05 ± 0.00 b
Cu (mg/kg)	7.71 ± 0.16 b	8.80 ± 0.21 a	4.24 ± 0.07 a	3.84 ± 0.73 a	3.60 ± 0.20 a	3.56 ± 0.13 a	3.98 ± 0.11 a	2.98 ± 0.70 a
Fe (mg/kg)	81.27 ± 6.24 a	43.07 ± 1.47 a	56.73 ± 2.59 a	47.43 ± 5.72 ab	48.95 ± 1.89 ab	44.51 ± 0.87 ab	54.70 ± 1.14 a	36.65 ± 7.27 b
Mn (mg/kg)	12.93 ± 0.01 b	22.48 ± 0.44 a	16.14 ± 0.47 a	14.72 ± 2.42 a	13.79 ± 0.33 a	13.51 ± 0.79 a	16.52 ± 0.79 a	5.47 ± 0.89 b
Zn (mg/kg)	27.20 ± 0.05 b	41.66 ± 0.87 a	16.36 ± 0.16 a	15.07 ± 2.16 ab	14.80 ± 0.73 ab	13.33 ± 0.40 ab	16.23 ± 1.07 a	11.07 ± 1.72 b
Antioxidant Activity and Total Phenolic Content								
FRAP (µmol TE/100 g)	0.19 ± 0.07 b	1.99 ± 0.07 a	1391.33 ± 166.95 a	1433.66 ± 160.95 a	1497.27 ± 142.10 a	1508.59 ± 46.80 a	1581.31 ± 59.00 a	490.22 ± 48.67 b
ABTS (µmol TE/100 g)	0.62 ± 0.01 b	2.74 ± 0.13 a	2266.20 ± 94.60 a	2334.31 ± 133.50 a	2249.41 ± 139.63 a	2472.46 ± 57.83 a	2330.95 ± 17.69 a	898.21 ± 55.36 b
DPPH (µmol TE/100 g)	0.78 ± 0.22 b	4.48 ± 0.78 a	3500.25 ± 131.18 ab	3468.07 ± 130.80 ab	3528.31 ± 104.22 ab	3669.69 ± 114.65 a	3513.50 ± 174.74 ab	3188.55 ± 66.62 b
TPC (mg GAE/100 g)	1.32 ± 0.07 b	2.19 ± 0.06 a	172.87 ± 1.26 a	162.82 ± 6.82 a	55.25 ± 26.30 b	160.06 ± 31.59 a	72.30 ± 48.40 b	36.24 ± 4.53 b

Results were expressed as the mean ± standard deviation. For mushroom by-products, different letters within the same row indicate statistically significant differences, as determined by Student’s *t*-test (*p* < 0.05). For each gluten-free cookie formulation, different letters within the same row indicate statistically significant differences, according to Tukey HSD (*p* < 0.05). Abbreviations: MC: control mushroom by-products; MU: UAE-treated mushroom by-products; CC: gluten-free control cookie; C-4MC: gluten-free cookie with 4% MC; C-4MU: gluten-free cookie with 4% MU; C-8MC: gluten-free cookie with 8% MC; C-8MU: gluten-free cookie with 8% MU; COM: commercial gluten-free cookie; IDF: insoluble dietary fiber; SDF: soluble dietary fiber; TDF: total dietary fiber; TE: trolox equivalents; TPC: total phenolic content; GAE: gallic acid equivalents.

**Table 3:** Physicochemical parameters of gluten-free cookies.

	CC	C-4MC	C-4MU	C-8MC	C-8MU	COM
CIEL*a*b* Parameters						
L*	28.51 ± 0.25 c	29.36 ± 0.12 bc	30.72 ± 0.45 bc	29.62 ± 0.25 bc	31.38 ± 0.10 b	67.61 ± 2.35 a
*C*	12.66 ± 0.25 b	16.18 ± 6.61 b	13.32 ± 0.55 b	12.81 ± 0.22 b	14.27 ± 1.03 b	34.71 ± 2.13 a
*h*	43.07 ± 0.35 d	44.13 ± 0.68 d	46.38 ± 0.63 c	45.99 ± 0.62 c	48.09 ± 0.49 b	73.30 ± 0.68 a
a*	9.25 ± 0.13 ab	9.01 ± 0.21 b	9.19 ± 0.33 ab	8.90 ± 0.16 b	9.53 ± 0.61 ab	9.96 ± 0.25 a
b*	8.65 ± 0.23 b	8.61 ± 0.21 b	9.65 ± 0.47 b	9.21 ± 0.21 b	10.62 ± 0.85 b	33.25 ± 2.15 a
∆E*	-	0.94 ± 0.22 b	2.48 ± 0.62 b	1.35 ± 0.19 b	3.61 ± 0.66 b	46.20 ± 2.80 a
Acidity Profile						
pH	5.46 ± 0.14 c	5.52 ± 0.04 bc	5.69 ± 0.06 abc	5.95 ± 0.18 a	5.70 ± 0.05 abc	5.81 ± 0.18 ab
TTA (ml NaOH/10 g)	9.10 ± 1.22 ab	7.90 ± 0.20 b	8.10 ± 0.19 ab	6.27 ± 0.18 c	9.42 ± 0.33 a	2.53 ± 0.36 d

a–d: Different letters within the same row indicate significant differences between samples (*p* <0.05). Abbreviations: MC: control mushroom by-products; MU: UAE-treated mushroom by-products; CC: gluten-free control cookie; C-4MC: gluten-free cookie with 4% MC; C-4MU: gluten-free cookie with 4% MU; C-8MC: gluten-free cookie with 8% MC; C-8MU: gluten-free cookie with 8% MU; COM: commercial gluten-free cookie; L*: lightness, *C*: chroma, *h*: hue, a*: green-red axis; b*: blue-yellow axis; ∆E*: Total color difference of each formulation compared to CC; TTA: total titratable acidity.

## Data Availability

The original contributions presented in this study are included in the article/Supplementary Materials. Further inquiries can be directed to the corresponding author.

## Supplementary Materials

The following supporting information can be downloaded at https://www.mdpi.com/article/10.3390/antiox15101347/s1, Table S1: Two-way ANOVA and simple effects of by-product treatment and inclusion level in mushroom-containing cookie formulations; Table S2: Mineral content of gluten-free cookies and mushroom by-products; Table S3: Putatively annotated compounds revealed in mushroom by-products and gluten-free cookies after untargeted UHPLC-QTOF-HRMS analysis; Table S4: Variable Importance in Projection (VIP) markers of mushroom by-products from supervised OPLS-DA of UAE treatment against control; Table S5: Discriminant accumulated metabolites (VIP score > 1, FC ≥ ±2 and *p* < 0.05) of mushroom by-products from supervised OPLS-DA and volcano analysis of UAE treatment against control; Table S6: ChemRICH results based on discriminant metabolites (VIP score > 1, log_2_ FC ≥ ±2 and *p* < 0.05) in mushroom by-products; Table S7: Variable Importance in Projection (VIP) markers of gluten-free cookies from supervised OPLS-DA of all treatments against control; Table S8: Discriminant accumulated metabolites (VIP score > 1, log_2_ FC ≥ ±2 and *p* < 0.05) of gluten-free cookies from supervised OPLS-DA and volcano analysis of all treatments against control. Figure S1: Permutation tests for OPLS-DA models comparing MU against MC (A), and C-4MC (B), C-4MU (C), C-8MC (D), and C-8MU (E), against CC; Figure S2: Chemometric analysis on the untargeted UHPLC-QTOF-HRMS metabolomic profiling of mushroom by-products.
