# Supplementary material for: Ultrasound-Assisted Extraction of Pleurotus ostreatus By-Products for the Development of Nutritionally Enhanced Gluten-Free Cookies: Nutritional, Sensory, and Metabolomic Insights
Source: Antioxidants (Basel). 2026 Oct 19;15(10):1347. doi: 10.3390/antiox15101347 (PMC13701189; doi:10.3390/antiox15101347)
Supplement: Supplementary file 1 [file antioxidants-15-01347-s001.zip › antioxidants-4608956-Supplementary Figures.pdf]

Supplementary Figures Caption

Supplementary Figure S1. Permutation tests for OPLS-DA models comparing MU against_MC **(A)**, and C-4MC **(B)**, C-4MU **(C),** C-8MC **(D),** and C-8MU **(E)**, against CC.

Supplementary Figure S2. Chemometric analysis on the untargeted UHPLC-QTOF-HRMS metabolomic profiling of mushroom by-products. **A.** Hierarchical cluster analysis (HCA) obtained according to fold change-based heatmap (Euclidean distance, Ward's clustering algorithm). The heat color scale represents the FC values for each feature with respect to the median value involving all treatments. **B.** Principal component analysis (PCA), R^2=0.538, PERMANOVA *p-value*=0.001. **C.** Hierarchical cluster analysis (HCA) obtained according to fold change-based heatmap (Euclidean distance, Ward's clustering algorithm) of CC and COM samples. The heat color scale represents the FC values for each feature with respect to the median value involving all treatments. **D.** Principal component analysis (PCA) of CC and COM samples, R^2=0.845, PERMANOVA *p-value*=0.003. **E.** Volcano plot ($\log_2$(FC) ≥ |±2|; *p-value* < 0.05) of the differential metabolites derived from CC compared to COM. **Abbreviations:** CC: gluten-free control cookie; C-4MC: gluten-free cookie with 4% MC; C-4MU: gluten-free cookie with 4% MU; C-8MC: gluten-free cookie with 8% MC; C-8MU: gluten-free cookie with 8% MU; COM: commercial .gluten-free cookie; PC1: first principal component; PC2: second principal component.

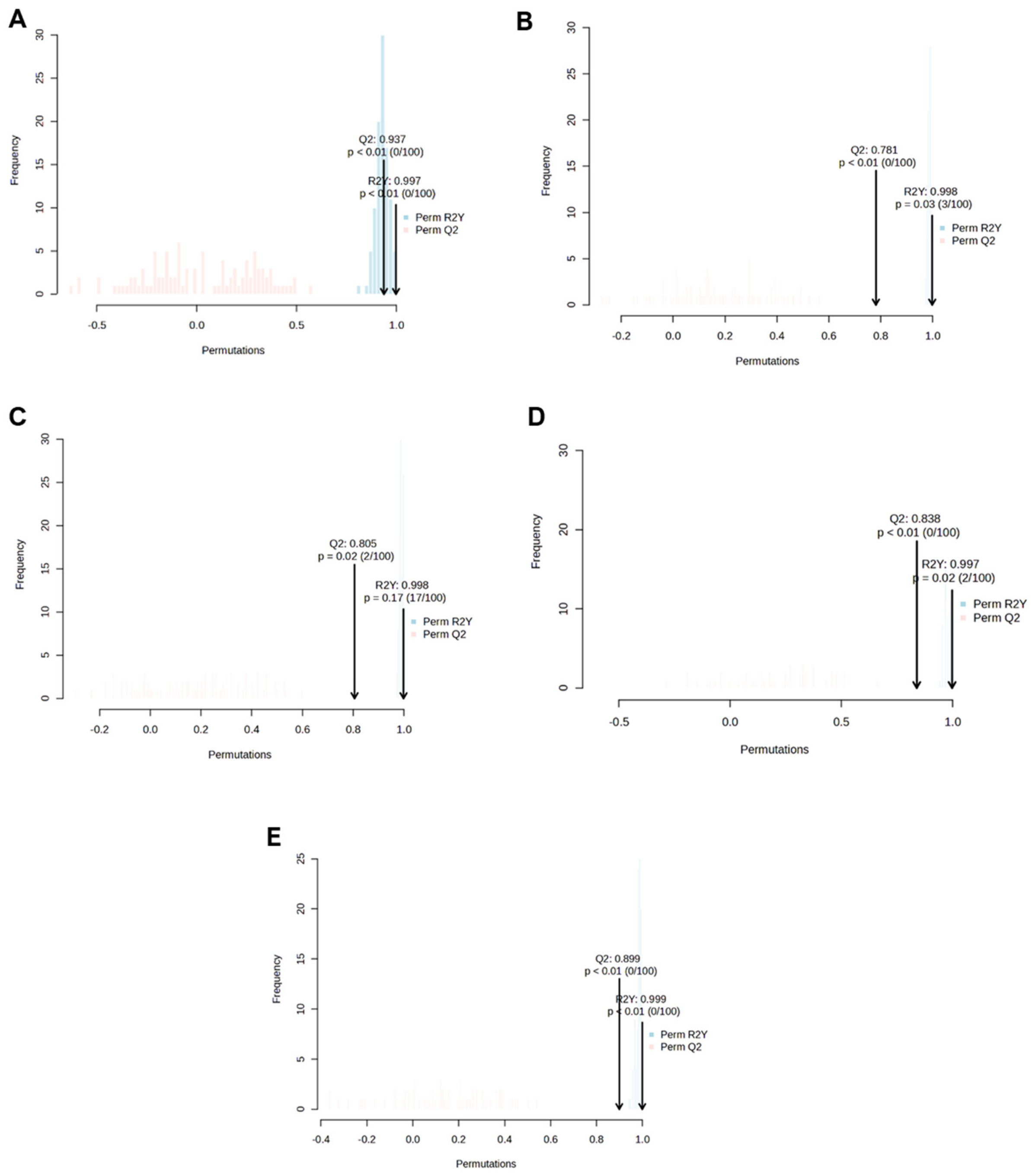

Supplementary Figure S1.

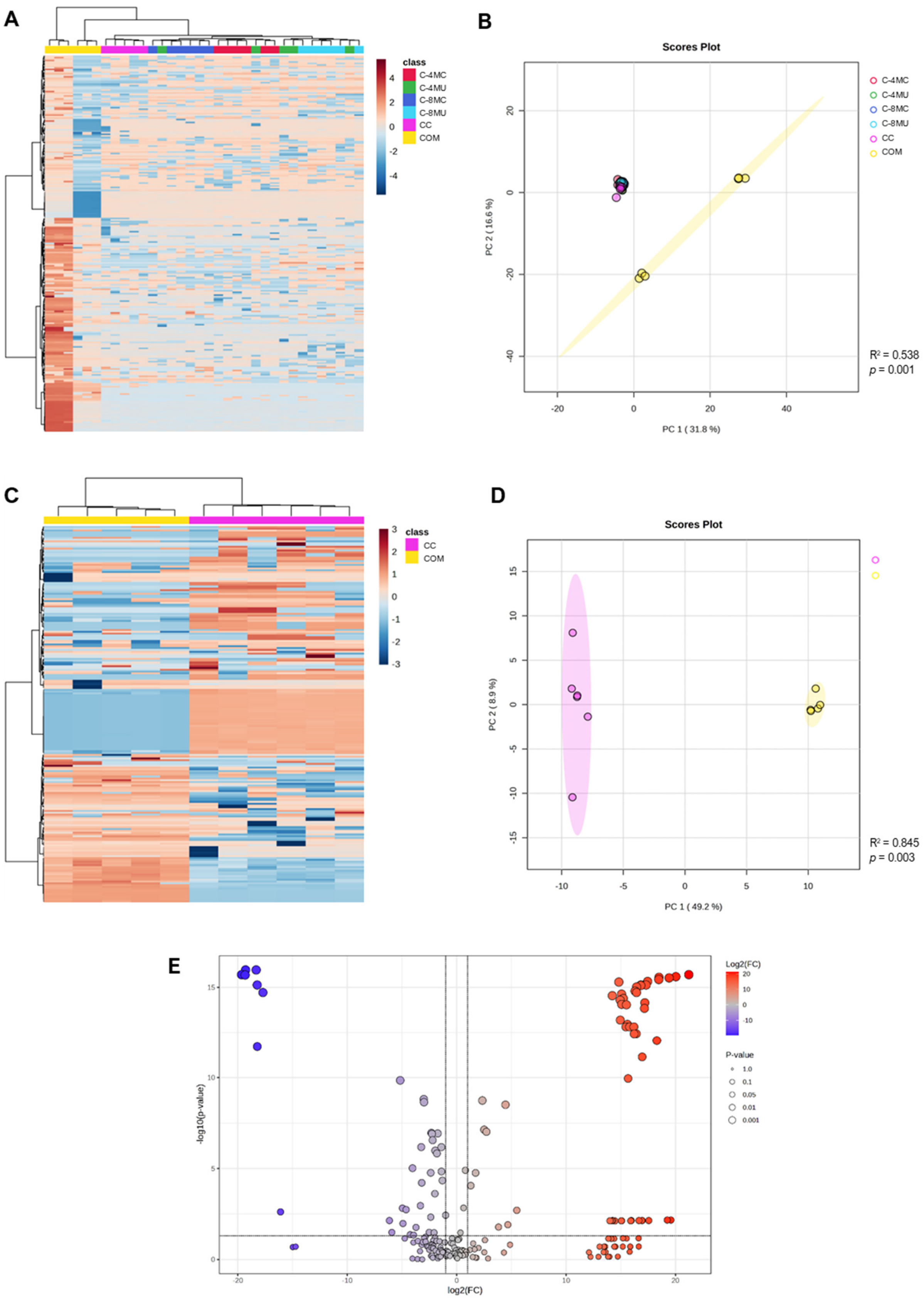

Supplementary Figure S2.
